# Supplementary material for: Photoswitchable paclitaxel-based microtubule stabilisers allow optical control over the microtubule cytoskeleton
Source: Nat Commun. 2020 Sep 15;11:4640. doi: 10.1038/s41467-020-18389-6 (PMC7493900; doi:10.1038/s41467-020-18389-6)
Supplement: Supplementary file 3 — Description of Additional Supplementary Files [file 41467_2020_18389_MOESM3_ESM.pdf]

Description of Additional Supplementary Files

File Name: Supplementary Movie 1

Description: (data corresponding to Fig 5): HeLa cells expressing EB3-tdTomato incubated with *E*-**AzTax3MP** (1 μM) were imaged at 561 nm according to the cell-specific isomerisation imaging protocol. The periods of 405 nm pulsed illuminations (each indicated by a blue filled circle) were applied to the cell indicated by the blue arrow, with intervals ca. 100 s between periods, illuminating a region of interest (ROI) drawn within the boundaries of the indicated cell (see also Fig 5a). This protocol caused cell-specific reductions of EB3 comet count and dynamics during the illumination periods, that recovered between periods to the level of the non-ROI cells analysed in the same field of view (indicated by the white arrows), that received identical *E*-**AzTax3MP** treatment but were not targeted for illumination at 405 nm.

File Name: Supplementary Movie 2

Description: (data corresponding to Fig 5; cosolvent control movie, to be compared to Supplementary Movie 1): EB3-tdTomato-transfected HeLa cells were imaged at 561 nm, and the blue arrow-indicated cell was illuminated at 405 nm, under identical conditions as in Supplementary Movie 1 but without **AzTax3MP** (cosolvent control experiment; for cell ROI boundaries see Fig 5a). This caused no cell-specific reductions of EB3 comet counts or dynamics.

File Name: Supplementary Movie 3

Description: (data corresponding to Supplementary Figure 5): mCherry-α-tubulin transfected COS-7 cells were imaged at 561 nm in the presence of 4 μM **AzTax3MP**. A blue circle indicates full frame illumination with 405 nm for the duration of 6 min. No significant reduction of existing microtubule structure was observed.

File Name: Supplementary Movie 4

Description: (data corresponding to Supplementary Figure 5, cosolvent control, to be compared to Supplementary Movie 3): mCherry-α-tubulin transfected Cos-7 cells were imaged at 561 nm in the presence of 1% DMSO. A blue circle indicates full frame illumination with 405 nm for the duration of 5 min starting at timestamp 03:00. A baseline for the microtuble structure was established.

File Name: Supplementary Movie 5

Description: (cosolvent control movie, to be compared to Supplementary Movie 6, data corresponding to Fig 6a): A primary neuron cell transfected with EB3-tdTomato and treated with 1% DMSO was imaged at 561 nm while applying 405 nm illuminations in the blue box (inset at top right shows magnified zoom). The movie was taken before Supplementary Movie 6 (both show the same primary neuron) to enable direct comparison. Kymographs were taken of the area labelled by the blue box (Fig 6).

File Name: Supplementary Movie 6

Description: (to be compared to Supplementary Movie 5, data corresponding to Fig 6a): The primary neuron imaged in Supplementary Movie 5 was then treated with **AzTax3MP** (0.5 µM) and imaged at 561 nm while applying 405 nm illuminations to the same indicated blue box, starting from timestamp 02:00 (inset shows magnification). Only after beginning illuminations are comets travelling through the illuminated area seen to decrease, indicating localised suppression of MT polymerisation dynamics. Kymographs of the blue box area are shown in Fig 6.

File Name: Supplementary Movie 7

Description: A separate primary neuron, treated and imaged entirely as in Supplementary Movie 5 (cosolvent control for baseline before **AzTax** application).

File Name: Supplementary Movie 8

Description: The primary neuron from Supplementary Movie 7 was then treated with **AzTax3MP** (0.5 µM) and imaged with ROI-localised 405 nm illuminations, entirely as in Supplementary Movie 6 (pre-photoswitching baseline until 02:00, then localised EB suppression after photoswitching).

File Name: Supplementary Movie 9

Description: Imaging of EB3-tagRFP-T-transfected HeLa cells (at 561 nm), treated with *E*-**AzTax3MP** (0.6 µM), while periods of full-frame 405 nm illuminations with higher photon flux are applied (indicated by blue dots); EB3 tip end comet count and dynamics progressively diminish during illumination while the marker begins to label the entire MT lattice, which becomes curled and contorted (see Supplementary Information for details and discussion).

File Name: Supplementary Movie 10

Description: (cosolvent control for Supplementary Movie 9): Cells prepared and imaged as in Supplementary Movie 9 but without **AzTax3MP** show no effects on microtubule dynamics or lattice structure.

File Name: Supplementary Movie 11

Description: TIRF imaging of EB3-tagRFP-T-transfected HeLa cells (at 561 nm), treated with *E*-**AzTax3MP** (1.0 µM), while periods of full-frame 405 nm illuminations (see Supplementary Information for details) are applied to isomerise it partially to *Z*. Upon light application MT dynamics cease and return in periods without 405 nm light (results comparable to Supplementary Movies 1-2). The change of observation technique allows for a different perspective on the observed effects.

File Name: Supplementary Movie 12

Description: (photoswitching control to be compared to Supplementary Movie 11): TIRF imaging of EB3-tagRFP-T-transfected HeLa cells (at 561 nm), treated with *E*-**AzTax3MP** (1 µM) with no 405 nm light application, to avoid photoswitching to *Z*. Only a slight change of the MT velocity can be observed (compare to Fig 5c).

File Name: Supplementary Movie 13

Description: (cosolvent and light control to be compared to Supplementary Movie 11): TIRF imaging of EB3-tagRFP-T-transfected HeLa cells (at 561 nm), treated with 1% cosolvent with the same light regime as movie 11. Under the 405 nm illumination regime, DMSO alone has no effect on MT dynamics.

File Name: Supplementary Movie 14

Description: (cosolvent control to be compared to Supplementary Movie 11): TIRF imaging of EB3-tagRFP-T-transfected HeLa cells (at 561 nm), treated with 1% cosolvent and imaged. DMSO alone has no effect on MT dynamics.
