# Supplementary material for: Photoswitchable paclitaxel-based microtubule stabilisers allow optical control over the microtubule cytoskeleton
Source: Nat Commun. 2020 Sep 15;11:4640. doi: 10.1038/s41467-020-18389-6 (PMC7493900; doi:10.1038/s41467-020-18389-6)
Supplement: Supplementary file 18 — Reporting Summary [file 41467_2020_18389_MOESM18_ESM.pdf]

nature research

Corresponding author(s): Oliver Thorn-Seshold
Last updated by author(s): 13/08/2020

Reporting Summary

Nature Research wishes to improve the reproducibility of the work that we publish. This form provides structure for consistency and transparency in reporting. For further information on Nature Research policies, see Authors & Referees and the Editorial Policy Checklist.

Statistics

For all statistical analyses, confirm that the following items are present in the figure legend, table legend, main text, or Methods section.

n/a	Confirmed	
☐	☒	The exact sample size (n) for each experimental group/condition, given as a discrete number and unit of measurement
☐	☒	A statement on whether measurements were taken from distinct samples or whether the same sample was measured repeatedly
☐	☒	The statistical test(s) used AND whether they are one- or two-sided<br>Only common tests should be described solely by name; describe more complex techniques in the Methods section.
☒	☐	A description of all covariates tested
☒	☐	A description of any assumptions or corrections, such as tests of normality and adjustment for multiple comparisons
☐	☒	A full description of the statistical parameters including central tendency (e.g. means) or other basic estimates (e.g. regression coefficient) AND variation (e.g. standard deviation) or associated estimates of uncertainty (e.g. confidence intervals)
☐	☒	For null hypothesis testing, the test statistic (e.g. F, t, r) with confidence intervals, effect sizes, degrees of freedom and P value noted<br>Give P values as exact values whenever suitable.
☒	☐	For Bayesian analysis, information on the choice of priors and Markov chain Monte Carlo settings
☒	☐	For hierarchical and complex designs, identification of the appropriate level for tests and full reporting of outcomes
☒	☐	Estimates of effect sizes (e.g. Cohen's d, Pearson's r), indicating how they were calculated

Our web collection on statistics for biologists contains articles on many of the points above.

Software and code

Policy information about availability of computer code

Data collection	Flow Cytometry data was collected using BD FACSDiva 8.0.1<br>Live cell imaging data collection was controlled using MetaMorph 7.7
Data analysis	Dose-response curves were fitted using GraphPad Prism version 8.3.0 for MacOS<br>Flow Cytometry data was gated and analyzed using FlowJo 10.6.0<br>Live cell imaging data/commet count was quantified using ImageJ ((FIJI; 2.0.0-rc-69/1.52p) using the ComDet plugin (E. Katrukha, University of Utrecht, https://github.com/ekatrukha/ComDet)

For manuscripts utilizing custom algorithms or software that are central to the research but not yet described in published literature, software must be made available to editors/reviewers. We strongly encourage code deposition in a community repository (e.g. GitHub). See the Nature Research guidelines for submitting code & software for further information.

Data

Policy information about availability of data

All manuscripts must include a data availability statement. This statement should provide the following information, where applicable:

- Accession codes, unique identifiers, or web links for publicly available datasets
- A list of figures that have associated raw data
- A description of any restrictions on data availability

Figures 2-5 and Supplemental Figures 1-5 have raw data associated that is presented in the source data file. This and all data of the study can also be obtained from the authors upon request. None of these datasets are resources of public interest and therefore are not archived publicly in other forms.

October 2018

Field-specific reporting

Please select the one below that is the best fit for your research. If you are not sure, read the appropriate sections before makingyour selection.

☒ Life sciences ☐ Behavioural & social sciences ☐ Ecological, evolutionary & environmental sciences

For a reference copy of the document with all sections, see nature.com/documents/nr-reporting-summary-flat.pdf

Life sciences study design

All studies must disclose on these points even when the disclosure is negative.

Sample size	No explicit sample size calculation was performed. At least three independent experiments were performed for quantifiable assays, according to the large expected effect size. All assays were performed in multiple concentrations, giving dose-response controls. All assays were performed in technical triplicates. This matches accepted practice (e.g. https://doi.org/10.1016/j.cell.2015.06.049)
Data exclusions	No data was excluded from the presented assays.
Replication	All relevant assays were done in independent biological replicates. All attempts at replication were successful.
Randomization	No randomisation was necessary, as starting conditions for all experiments are the same in the cellular assays.
Blinding	Blinding was not performed as assay readout is mostly unbiased (plate reader and flow cytometry). Microscopic evaluation was performed independently by two separate scientists.

Reporting for specific materials, systems and methods

We require information from authors about some types of materials, experimental systems and methods used in many studies. Here, indicate whether each material, system or method listed is relevant to your study. If you are not sure if a list item applies to your research, read the appropriate section before selecting a response.

Materials & experimental systems

n/a	Involved in the study	
☐	☒	Antibodies
☐	☒	Eukaryotic cell lines
☒	☐	Palaeontology
☐	☒	Animals and other organisms
☒	☐	Human research participants
☒	☐	Clinical data

Methods

n/a	Involved in the study	
☒	☐	ChIP-seq
☐	☒	Flow cytometry
☒	☐	MRI-based neuroimaging

Antibodies

Antibodies used	rabbit anti-alpha Tubulin antibody ab18251 (abcam)<br>donkey-anti-rabbit Alexa488 A21206 (Thermo Fisher Scientific)
Validation	no own validation performed; the manufacturers' websites cite validating publications (https://www.abcam.com/alpha-tubulin-antibody-ab18251.html and https://www.thermofisher.com/antibody/product/Donkey-anti-Rabbit-IgG-H-L-Highly-Cross-Adsorbed-Secondary-Antibody-Polyclonal/A-21206)

Eukaryotic cell lines

Policy information about cell lines

Cell line source(s)	HeLa (ATCC CCL-2), COS-7 (ATCC CRL-1651)
Authentication	cell lines were not authenticated (authentication is not essential for this study, as we are examining basic biological processes that are independent of cell type)
Mycoplasma contamination	cell lines were regularly tested for mycoplasma contamination. only mycoplasma negative cell lines were used in this study
Commonly misidentified lines<br>(See ICLAC register)	no commonly misidentified cell lines were used in this study

Animals and other organisms

Policy information about studies involving animals; ARRIVE guidelines recommended for reporting animal research

Laboratory animals	Wistar rats (Janvier): at least 10 weeks of age (female), embryonic day 18 pups (male and female).
Wild animals	No wild animals
Field-collected samples	No Field collected samples
Ethics oversight	All animal experiments were approved by the Dutch Animal Experiments Committee (DEC, Dier Experimenten Commissie) and were in line with the institutional guidelines of Utrecht University. Animal license number AVD1080020173404.

Note that full information on the approval of the study protocol must also be provided in the manuscript.

Flow Cytometry

Plots

Confirm that:

☒ The axis labels state the marker and fluorochrome used (e.g. CD4-FITC).

☒ The axis scales are clearly visible. Include numbers along axes only for bottom left plot of group (a 'group' is an analysis of identical markers).

☒ All plots are contour plots with outliers or pseudocolor plots.

☒ A numerical value for number of cells or percentage (with statistics) is provided.

Methodology

Sample preparation	HeLa cells were seeded in 6 well plates (300,000/well) 24 h prior to treatment. AzTax3MP and AzTax4DMA were added to the wells and cells were incubated either under "dark" or "lit" regimens.
Instrument	LSR Fortessa (BD Biosciences)
Software	Data collection: BD FACSDiva 8.0.1<br>Data analysis: FlowJo software (BD Biosciences)
Cell population abundance	1) gating for living cells 85%<br>2) single cell gating 98%
Gating strategy	1) FSC-A vs. SSC-A<br>2) FSC-H vs. FSC-W<br>3) PI gating for cell cycle

☒ Tick this box to confirm that a figure exemplifying the gating strategy is provided in the Supplementary Information.
